# Postoperative Ascites of Unknown Origin following Laparoscopic Appendicectomy: An Unusual Complication of Laparoscopic Surgery

**DOI:** 10.1155/2014/549791

**Published:** 2014-04-13

**Authors:** M. Feretis, H. Boyd-Carson, A. Karim

**Affiliations:** Department of General Surgery, Heart of England NHS Foundation Trust, Birmingham, UK

## Abstract

Postoperative ascites is a very rare complication of laparoscopic surgery. Significant iatrogenic injuries to the bowel, the urinary tract, and the lymphatic system should be excluded promptly to avoid devastating results for the patient. In some cases, in spite of investigating patients extensively, no definitive causative factor for the accumulation of fluid can be identified. In such cases, idiopathic allergic or inflammatory reaction of the peritoneum may be responsible for the development of ascites. We present a case of ascites of an unknown origin in a young female patient following a laparoscopic appendicectomy.

## 1. Introduction

Increasing use of laparoscopy over the past 3 decades has led to an increasing number of reports on adverse events attributed to such procedures. The most commonly reported complications include visceral injury (bowel/urinary tract), vessel injury, gas embolism, and subcutaneous emphysema [1].

The development of ascites after laparoscopic surgery has multiple aetiologies (i.e., unrecognised bowel or urinary tract injury), each requiring a different treatment strategy. However, in a small number of patients no causative factor can be identified for the development of ascites despite investigating those patients extensively.

We present a rare complication of laparoscopic surgery, that of ascites of unknown origin following laparoscopic appendicectomy in a young female patient.

## 2. Case Report

A twenty-five year old Caucasian female was admitted acutely with right iliac fossa (RIF) pain and maximal tenderness over McBurney's point. Past medical history included sciatica and cervical intraepithelial neoplasia grade III. There was no previous surgical history of note.

Admission blood tests revealed a haemoglobin of 136 g/L (range 135–160 g/L), white cell count of 16.69 × 10^9^/L (4.0–11.0 × 10^9^/L), neutrophil count of 11.51 × 10^9^/L (2.0–7.5 × 10^9^), a serum amylase of 16 iu/L (25–125 iu/L), and a C-reactive protein (CRP) of 194 mg/L (0–5 mg/L). Serum liver and renal function tests on admission and were within normal reference range. Working diagnosis was acute appendicitis and the patient underwent an emergency laparoscopic appendicectomy.

## 3. The Procedure

Pneumoperitoneum was established using the open technique (12 mm Hg). All ports were inserted under direct vision. A 10 mm infraumbilical port was initially inserted followed by a further 10 mm port in the left iliac fossa (LIF) and a 5 mm suprapubic port. Laparoscopy was performed and the findings were as follows: no free intraperitoneal fluid was noted, the upper abdominal viscera looked unremarkable, and the appendix looked macroscopically normal. Inspection of the pelvis did not reveal evidence of free fluid; however, expert's opinion was sought from a gynaecologist with regard to the appearance of the right ovary (fallopian cysts seen). Decision was made that no intervention was required with regard to the incidental gynaecological finding and the surgical team proceeded with removal of the appendix. The appendicectomy was performed uneventfully with the use of electrosurgical surgery. The patient was transferred to the ward for routine postoperative care.

### 3.1. Postoperative Period

On day 1 post op, 18 hours following the procedure, the patient developed lower abdominal discomfort. On examination, she was apyrexial with no haemodynamic compromise. The patient's urine output (voluntary voiding) had been recorded as “adequate” on the observation charts. Palpation of the abdomen did not reveal signs of peritonism; however, significant infraumbilical swelling was noted and the diagnosis of subcutaneous wound haematoma was established. Performed serum laboratory investigations revealed a haemoglobin of 125 g/L and a white cell count of 18.4 × 10^9^/L. Renal function remained within the normal reference range and a urine dipstick test was negative for urological pathology. At 26 hours post op, the patient complained of increasing abdominal distension and worsening pain. On examination, the initial swelling had progressed to widespread abdominal distension; the abdomen remained soft. The decision was made to proceed with wound exploration under anaesthetic.

In theatre, wound exploration did not reveal evidence of subcutaneous or rectus sheath haematoma. However, clear fluid was noted to be exuding from the site of rectus sheath closure. The rectus sheath wound was opened and 2 litres of haemoserous fluid was suctioned out. Diagnostic laparoscopy was then undertaken and a further 1 L of fluid was removed. Inspection of the right iliac fossa and pelvis was unremarkable. The operating team proceeded to a diagnostic laparotomy through a low midline incision. Careful inspection of the reproductive organs, the bladder, the stomach/liver/gallbladder, the colon, and the small bowel did not reveal signs of iatrogenic injury or abnormality. In total, 3 litres of haemoserous intraperitoneal fluid was removed. Subsequently, thorough lavage of the peritoneal cavity was performed using 0.9% saline and a drain was left in place in the patient's pelvis. The procedure was completed uneventfully and the patient was commenced on intravenous antibiotics (coamoxiclav and metronidazole).

Biochemical analysis of fluid drained from the abdomen revealed a white blood cell count of 40/uL, albumin of <4 g/L, protein of 9 g/L, and LDH of 374 U/L. Fluid cultures for aerobic/anaerobic organisms and* Mycobacterium tuberculosis* did not grow any organisms and no malignant cells were noted on cytology. Histological analysis of the appendix revealed a luminal faecolith with no evidence of inflammation or malignancy.

Postoperatively, there were no complications, the drain output was minimal, and the patient was discharged on day 3 after an uneventful recovery period. No further readmissions were required and on 2-month follow-up no further adverse events or reaccumulation of fluid were reported.

## 4. Discussion

This was an unusual case of ascites developing within 24 hrs of laparoscopic appendicectomy for a noninflamed appendix with no evidence of peritoneal contamination. No definitive causative factor was identified in spite of performing a thorough postoperative biochemical and cytological analysis of the accumulated fluid. Following exploratory laparotomy and drainage of the accumulated fluid, no further intra-abdominal fluid accumulation occurred without further therapeutic intervention being given to the patient other than intravenous antibiotics. We attribute the development of ascites to an idiopathic allergic or inflammatory peritoneal reaction to the laparoscopic procedure.

The primary concern during exploratory laparotomy was to exclude a significant complication (bowel/urinary tract injury) that could have been caused by the initial laparoscopic procedure. Furthermore, we investigated the unexpected fluid accumulation further to exclude other causes of ascites (i.e., acute pancreatitis) that might not have been associated directly with the initial procedure.

Bowel injury is a common cause of postoperative peritoneal fluid accumulation following a laparoscopic procedure. In the literature, the estimated incidence of bowel injury is reported to be 0–0.5% with approximately half of the injuries occurring during entry into the peritoneum [1]. It is one of the most important complications of laparoscopic surgery as it is potentially life-threatening if not identified at the time of the operation [2–4]. If intraoperatively unrecognised, patients often develop nonspecific complaints such as fever, diarrhoea, and abdominal distension/pain, not exactly pointing to peritonitis leading to further delay of establishing the diagnosis [5]. In our patient, the laparoscopic ports were inserted under direct vision; the peritoneal fluid had a clear colour on exploratory laparotomy and fluid cultures were negative. Inspection of the large and small bowel was normal; therefore, bowel injury was an unlikely the cause for the development of ascites.

Iatrogenic injuries to the urinary tract represent a significant complication of surgery, especially following laparoscopic procedures [6, 7]. They are common causes of large postoperative fluid accumulation in the peritoneal cavity [8]. The estimated rate of bladder injuries ranges from 0.02 to 8.3%, with the majority of injuries occurring during hysterectomy operations. Intraoperative identification of such injuries can be challenging for the surgeon, with approximately half of the injuries not being recognised at the time of surgery [9]. Further assessment of the urinary tract in cases of suspected injury can be performed by means of retrograde pyelography or excretory urograms and serum urea and creatinine measurements [10]. The colour of the peritoneal fluid, the thorough inspection of the bladder during exploratory laparotomy, the absence of microscopic haematuria and the nonelevated serum creatinine level effectively ruled out the possibility of iatrogenic urinary tract injury in our patient.

Collection of chyle in the peritoneal cavity as a result of lymph duct injury is another complication of laparoscopic surgery especially in cases of extensive retroperitoneal surgery [11–13]. In such cases, the peritoneal fluid has a distinctive appearance attributed to its high lipid content. Such diagnosis was considered unlikely by the authors regarding this particular patient as the retroperitoneal space was not entered during the procedure and the biochemical composition of the ascitic fluid was not suggestive of chyle.

Acute pancreatic ascites was another possible explanation for the development of ascites in our patient; however, the low levels of serum amylase and ascetic-fluid protein essentially ruled out such diagnosis.

To date, evidence in the literature to suggest the possibility of peritoneal allergic or inflammatory reaction to agents used during laparoscopic surgery in cases where visceral injury or other pathology has not been identified is limited to isolated case reports [14–17]. All reports on the subject have been produced on patients undergoing gynaecological procedures. After performing a systematic search on MEDLINE and EMBASE, we did not identify any previous reports on the development of postoperative idiopathic ascites following laparoscopic appendicectomy or other gastrointestinal surgery. Previous reports have suggested the possibility of allergic reaction to chemical agents used during laparoscopy (antiseptic peritoneal lavage and methylene blue dye) [16, 17]. However, our patient was not administered any specific drugs during the operation and the colour of the ascites was such that made the diagnosis of bacterial ascites unlikely. This was supported by the negative fluid cultures. We speculate that some substances used during laparoscopy (carbon dioxide, light/heat, diathermy) have triggered an inflammatory response explained by the elevated white cell count and the moderate hypoproteinaemia of the ascitic fluid.

## 5. Conclusion

Postoperative ascites of unknown origin after laparoscopic appendicectomy is a rare complication. Patients should be thoroughly investigated and monitored in order to exclude the possibility of an iatrogenic visceral injury during laparoscopy. Emergency laparotomy should be considered early, if the patient is developing signs of peritonitis. If no definitive cause for the ascites can be identified, the most likely explanation for the ascites is peritoneal inflammatory reaction to agents used during laparoscopy. In our experience, after draining the ascites, such patients recover well and no further intervention is required.
